# Regularities in human mortality after age 105

**DOI:** 10.1371/journal.pone.0253940

**Published:** 2021-07-14

**Authors:** Jesús-Adrián Alvarez, Francisco Villavicencio, Cosmo Strozza, Carlo Giovanni Camarda

**Affiliations:** 1 Interdisciplinary Centre on Population Dynamics, University of Southern Denmark, Odense, Denmark; 2 Department of International Health, Bloomberg School of Public Health, Johns Hopkins University, Baltimore, MD, United States of America; 3 Department of Statistical Sciences, Sapienza University of Rome, Rome, Italy; 4 Mortality, Health and Epidemiology Unit, National Institute for Demographic Studies (INED), Aubervilliers, France; Universidade Federal de Minas Gerais, BRAZIL

## Abstract

Empirical research on human mortality and extreme longevity suggests that the risk of death among the oldest-old ceases to increase and levels off at age 110. The universality of this finding remains in dispute because of two main reasons: i) high uncertainty around statistical estimates generated from scarce data, and ii) the lack of country-specific comparisons. In this article, we estimate age patterns of mortality above age 105 using data from the International Database on Longevity, an exceptionally large and recently updated database comprising more than 13,000 validated records of long-lived individuals from eight populations. We show that, in all of them, similar mortality trajectories arise, suggesting that the risk of dying levels off after age 105. As more high-quality data become available, there is more evidence in support of a levelling-off of the risk of dying as a regularity of longevous populations.

## Introduction

Several regularities are associated with human mortality. It is broadly accepted, for instance, that the risk of death starts growing exponentially with age at early adulthood [1]. However, at the frontier of human longevity, we are challenged by the limits of demographic knowledge. The trajectory of the risk of dying at extreme old ages is one of the demographic patterns that is most often questioned in evolutionary theories of aging [2–6] mathematical models [7–9] and empirical studies [10–14]. The identification of regularities in mortality patterns among the oldest-old has profound implications for societies and health sciences [15], and it can radically reshape evolutionary thinking [16]. In this article, we examine an unprecedentedly large and reliable dataset of the longest-lived individuals to evaluate whether a universal pattern of human longevity emerges at extreme old ages, thereby, providing new insights into and a better understanding of the biological mechanisms of human aging.

Previous empirical research on extreme longevity has led to opposing standpoints. Some scholars have suggested that there is a mortality plateau after age 110, estimating a constant risk of death of 0.70 after that age, which corresponds to an annual probability of dying of about 0.5 [10, 11]. Those results, however, have been questioned, based on: i) high uncertainty around the estimates, given the small number of observations (usually not exceeding 1,000 records), and ii) on the aggregation of populations, thereby masking regional and country specificity [12, 14]. Recently, a sample of 3,836 Italian semi-supercentenarians (individuals aged 105 years or older) has been used to show that the risk of dying reaches or closely approaches a plateau after age 105 [17]. This is an important result because it demonstrates a mortality plateau for a single country. Skepticism about the finding still prevails, however; mainly based on the statistical model chosen to evaluate the trajectory of the risk of dying [18–20].

As people live longer, and more individuals reach exceptionally advanced ages, larger datasets on the oldest-old become available. Key among them is the International Database on Longevity (IDL), which is the result of an unprecedented collaboration between statistical offices from different countries, demographers, gerontologists and experts on longevity [21]. The database, which was first launched in 2010, provided detailed information on 672 exhaustively validated cases of supercentenarians (individuals aged 110 or older) from a number of countries [22]. In 2020, the IDL was updated and it now provides additional high-quality data on individuals who survived to age 105, increasing the sample size to more than 13,000 records [23].

In this article, we examine data from eight populations included in the IDL that report validated records of all individuals aged 105 and above: France, Germany, Belgium, the United States, Denmark, Quebec, Austria and Norway. Apart from Quebec, all of them are nationally representative. For the United States, we start the analysis at age 110 because available data before that age is not country-representative [23]. We use a fully non-parametric framework to estimate the age trajectories of the risk of dying (see Materials and Methods for details). Although comparisons are more meaningful among countries with larger populations, the estimation of the risk of dying (i.e. mortality hazard) is still possible in the eight populations studied. Differences in population sizes are reflected in the size of the confidence intervals of the estimated hazards.

Our analyses represent three major contributions. First, the uncertainty around the estimates of the risk of dying is substantially reduced, by virtue of the large amount of high-quality data included in the analysis. Second, the exceptionally large dataset enables us to assess, for the first time, country-specific trajectories in the risk of dying above age 105. Third, our estimates are purely data driven; no model structure is imposed on the age patterns of mortality. These are three compelling features of our study that were neither achieved in previous studies of supercentenarians [12, 14], nor applied to datasets where the scarcity of data was overcome by aggregating populations [10, 11]. According to the hypothesis that there is a mortality plateau at extreme ages [10, 17], a leveling-off in the risk of dying is expected to arise as a regularity in all the populations analyzed.

## Results

**Fig 1:**
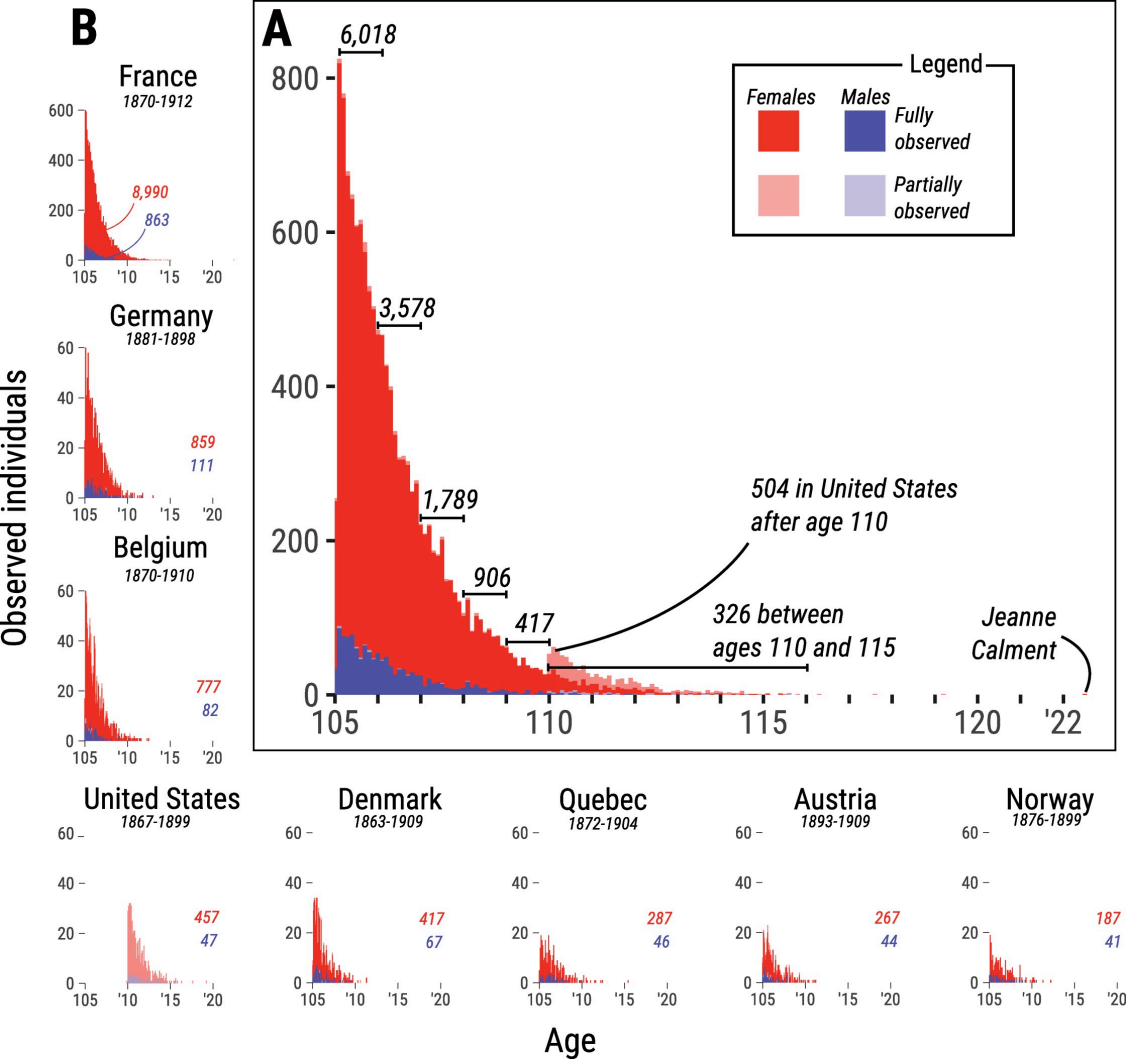
Histograms of individuals who reached age 105 reported in the International Database on Longevity, disaggregated by sex and observational scheme. (**A**) Histogram depicting the distribution of ages at last observation for all countries combined. (**B**) Close up to the distribution of ages at last observation by country and region. Red bars represent females and blue bars represent males. When the exact age at death is known, we regard an individual as fully observed and it is represented by solidly colored bars. Conversely, partially observed individuals are those whose exact date of death is unknown (e.g., right-censored or interval-censored) and they are depicted with lighter-colored bars. Birth cohorts of individuals included in the analysis are indicated below each country name.

Fig 1A shows the ages at last observation for all individuals included in the analysis. A rapid decrease in the number of individuals over age is observed: from 6,018 observations between ages 105–106 to 326 individuals above age 110, up to the longest documented lifespan–achieved by Jeanne Calment–who died at the age of 122 years and 164 days [24]. This pattern suggests an exponential decline in the number of survivors, which might thus call for an underlying constant risk of dying (i.e. mortality plateau). However, much heterogeneity is hidden in the main histogram of Fig 1A, because the number of observations is not evenly shared by countries and sexes. Females, for example, account for 90% of the data.

The insets of Fig 1B provide details on the distribution of observations by country. France accounts for the largest share in the IDL, with 9,853 observations (8,990 females and 863 males) from birth cohorts 1870–1912. Germany and Belgium show 970 and 859 observations, respectively, which represent about one-tenth of the population size in France. The United States also exhibits a large population size (504 individuals above age 110); however, all of its records are only partially observed, given that the exact dates of birth are not reported for confidentiality reasons [23]. We performed non-parametric imputation of birthdates to produce mortality estimates for the United States (see Materials and Methods). Finally, Denmark, Quebec, Austria and Norway each account for 200–400 observations. Despite the fact that the populations analyzed are all of different sizes, the distributions of individuals by age are similar across countries (Fig 1B). This indicates that a rapid decrease in the number of individuals over age, shown in Fig 1A, is a regularity of longevous populations.

**Fig 2:**
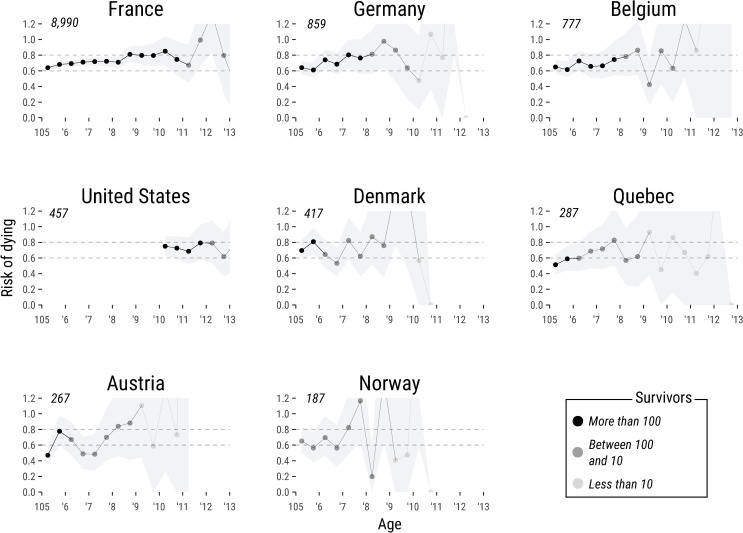
Risk of dying between ages 105 and 113 with 95% empirical confidence intervals for females. The estimation of the risks of dying and empirical confidence intervals was performed using a fully non-parametric approach and by considering the observations schemes in each country. The risk of dying is expressed in age intervals of six months. Population size is indicated at the top-left corner of each panel. The darker the shade of the dot plot, the greater the number of survivors at every specific age (i.e. subjects exposed to the risk of dying). For example, at age 105, there were 8,990 French females exposed to the risk of dying. At age 113, only 18 survived.

Fig 2 displays the risk of dying from ages 105 to 113 and 95% empirical confidence intervals (CI) for females. The risk of dying is expressed in age intervals of six months. In France, the risk of dying is 0.64 (95% CI 0.62–0.67) at age 105, and slowly increases toward values of about 0.8 at age 110. The large number of documented cases narrows confidence intervals between ages 105 and 110. The low uncertainty around the country-specific estimations of the risk of dying around these ages was never achieved in any previous study of extreme longevity [10, 11, 17].

A similar pattern to that found in France is replicated for the other female populations: the risk of death ranges between 0.6 and 0.7 at age 105 and goes up slightly, approaching values of around 0.8. These regularities are more evident in Germany and Belgium, where the number of observations is considerably large. In the United States, the risk of dying also oscillates between 0.6 and 0.8 after 110. Estimates for Denmark, Quebec, Austria and Norway also fluctuate between 0.6 and 0.8 from ages 105–110. However, confidence intervals in these populations are wider, because the number of observations is much smaller than in the other countries (e.g., France or Germany).

For males, the estimation of the risk of dying was only possible in France. As shown in S1 Table in S1 File, mortality patterns between 105 and 110 in French males are somewhat similar to the ones found in female populations. Male survivors older than 105 years in the other populations are too few (less than 100) to produce reliable estimations. Similarly, in any population (females and males), the number of individuals aged 113 and above are too few to produce reliable estimates. The lack of observations hinders the assessment of the age pattern of mortality past this age [25].

Finally, we performed two sensitivity analysis to test the robustness of our results. First, we reduced the age interval from six to three months (see S1 Fig in S1 File). The trajectories of the risk of dying are very much alike in both cases. This indicates that our results do not depend on the size of the age interval. Second, we calculated the risk of dying for the total population (i.e. adding up data for males and females, see S2 Fig in S1 File). The trajectory of the risk of dying for the total population is almost identical to the one depicted by females, which account for most of the observed cases. This analysis entails that the results shown in Fig 2 clearly depict the trajectories of the risk of dying above age 105 for all the populations analyzed here. Our results are also coherent with the age-pattern of risk of dying occurring prior age 105 (see S3 Fig in S1 File).

## Discussion

While it is true that population size and observation schemes play an important role in reducing the uncertainty around mortality estimates [10], our findings provide evidence of regularities in the age pattern of mortality after age 105: none of the populations analyzed in this study indicate a rapid increase in the mortality hazard after age 105.

Our results are in stark contrast to previous studies that argue that the levelling-off in the risk of death among the oldest-old is only observed when less accurate data are analyzed, and that more recent and reliable data depict a steady increase in the mortality risk [12, 14]. Indeed, data from recent cohorts (e.g. after 1910) are not included in the analysis for most of the countries because they are not yet available through the IDL [21]. We foresee that the inclusion of such data would have increased considerably the number of records, thereby, reducing the uncertainty in the estimates of the risk of dying in populations were confidence intervals are large.

A wide range of theoretical models from various mathematical perspectives (e.g., Markov chains, Weiner processes, frailty models, directionality theory and other stochastic matrix models) have been proposed to describe the mechanisms behind mortality plateaus that were previously found in several species [2–9, 20, 26]. A common factor among these models is their support for the asymptotic convergence of the risk of dying toward a constant at the extreme end (i.e. mortality plateau). The asymptotic convergence implies that the observed risk of dying might never be completely flat, but it might be that minuscule increases indicate proximity to the asymptotic mortality plateau [7, 8, 26–28]. The results of the present study are in line with the asymptotic behavior suggested by theoretical models, which contributes to bridging the demographic knowledge gap and help to enhance our understanding about mortality plateaus in human populations.

Another question that arises from our results is whether the levelling-off is immutable or flexible. This has key implications for human longevity prospects, because forecasts that propose that life expectancy might increase to more than 100 years hinge on reductions in the risk of death for centenarians and semi-supercentenarians [29]. In the light of our findings, we foresee two contrasting scenarios. In the first of these, if the levelling-off shown in our findings is immutable over time, the risk of death cannot be reduced after age 105. Thus, there might be a limit to life expectancy that will be approached as more individuals survive to advanced ages. In the second scenario, if the onset age of the levelling-off is shifted toward ages beyond 105 (e.g. 115), old-age deaths can be postponed toward even older ages and life expectancy can continue to increase. This scenario is plausible as it has been shown that gains in life expectancy in the last two centuries were achieved by shifting deaths from early to late ages [30, 31]. However, it remains to be seen whether medical advances and the biological plasticity of aging will continue their interplay, thus continuing to advance the frontier of human longevity.

## Materials and methods

### Mortality data

Exact dates of birth and death of all individuals who attained age 105 were obtained from the International Database on Longevity (IDL), which collects information from twelve European countries, Canada (Quebec only), Japan and the United States (see ref. [25] for detailed description of the database). The IDL is an open access database and it can be retrieved from https://www.supercentenarians.org/. The data used in this study were retrieved on December 1^st^, 2020.

When the date of death is not available, the IDL provides information on the last date an individual was confirmed to be alive. In the case of the United States, the exact dates are unknown: for each individual, only the years of birth and death together with the age (in days) are given.

Several sample frames are observed in the IDL, depending on the country [10, 22, 23]. The information is censored if we only know that the individual survived to a certain age (right-censored) or died between two ages (interval-censored, as in the United States). An observation is truncated if the individual was selected into the sample only because they survived to age 105 (left-truncated), or because they died before reaching a particular age (right-truncated).

### Estimation of the risk of dying

Let *X* be a random variable that denotes the length of life after age 105. Given the exact dates on which individuals were last observed (dead or alive), we were able to partition the data set into *J* age intervals of length δ. By taking into account the observation frames described above, we computed the number of events _*δ*_E_*x*_ and the exposures *_δ_N_x_* (number of subjects exposed to the risk of dying) within each age interval [*x,x+δ*) for *x*∈*I*≔{105,105+*δ*,…,105+*J*⋅*δ*}. Then we computed *_δ_M_x_* = _*δ*_E_*x*_/_*δ*_N_*x*_, i.e. the corresponding central death rate within each interval. By taking a small *δ, _δ_M_x_* provides a quasi-continuous estimate of the risk of dying *μ*(*x*).

### Estimation of confidence intervals

Because of the small number of observations at the highest ages, standard methods that rely on the central limit theorem to compute confidence intervals are not valid. Hence, we assessed uncertainty surrounding our estimates by computing 95% empirical confidence intervals from data simulation. First, assuming the risk of death is constant within each age interval, for each population we transformed the estimated death rates into probabilities _δ_q_x_ = 1−exp[−∑_*i∈I,i≤x*_ *δ⋅_δ_M_i_*]. Then, we simulated *n* individuals who die according to the corresponding *_δ_q_x_*. The values of *n* match the number of records in the IDL for each country or region. We replicated this exercise 10,000 times, obtaining, for each population, 10,000 sets of death probabilities over age. Finally, we back transformed these sets of probabilities into death rates, and calculated the corresponding confidence intervals.

### Random imputation of dates of birth in the United States

To use the same framework for the estimation of a quasi-continuous risk of dying in the United States, we carried out a random imputation of dates of birth. For all individuals, we first established lower and upper bounds of birth depending on the corresponding year of birth, year of death, and exact age at death (in days). For example:

- An individual born in 1900 and dying in 2010 at the exact age 109 years and 200 days could have been born on June 15, 1900 at the earliest, supposing they died on January 1, 2010 at that exact age. The latest date of birth is December 31, 1900, supposing they died on August 19, 2010 with 109 years and 200 days. This individual could not have died later, otherwise they would have turned out 110, since the year of birth is 1900.
- Besides, an individual born in 1900 and dying in 2010 at the exact age 110 years and 20 days could have been born on January 1, 1900 at the earliest, supposing they died on January 21, 2010 at that exact age. The latest date of birth is December 11, 1900 supposing they died on December 31, 2010 with exactly 110 years and 20 days.

Given these individual lower and upper bounds, we randomly imputed an exact date of birth to each US record. We then calculated the corresponding exact date of death given the exact age at death, which is known. We repeated the birthdate imputation ***m* = 1,000** times, and in each step, we computed ***k* = 10,000** sets of death probabilities over age, as for the other populations. We then aggregated the sets of all the imputation steps, obtaining ***m***×***k*** sets of death probabilities over age. We finally estimated the risk of dying over age from these ***m***×***k*** sets and corresponding empirical confidence intervals, as described above. These confidence intervals measure the uncertainty of the point estimates, also assessing the additional uncertainty due to the data imputation.

### Replicability of the results

We carried out all our analyses using the open-source statistical software R [32]. The R code and data to replicate all the results and figures presented here are available in the public repository https://github.com/jssalvrz/Mortality105.

## Supporting information

S1 File (DOCX)
