# Supplementary material for: Regularities in human mortality after age 105
Source: PLoS One. 2021 Jul 14;16(7):e0253940. doi: 10.1371/journal.pone.0253940 (PMC8279393; doi:10.1371/journal.pone.0253940)
Supplement: S1 File — (DOCX) [file pone.0253940.s001.docx]

**S1 File:**

**Regularities in human mortality after age 105**

Jesús-Adrián Alvarez*, Francisco Villavicencio, Cosmo Strozza, and Carlo Giovanni Camarda

*Correspondence: [alvarez@sdu.dk](mailto:alvarez@sdu.dk)

# Additional tables and figures

**S1 Table.** **Estimates of the risk of dying and 95% confidence intervals for males in France**

|  | **France** | |
| --- | --- | --- |
| **Age** | $\boldsymbol{\mu}\left( \boldsymbol{x} \right)$ | **95% CI** |
| 105.0 | 0.76 | (0.67,0.85) |
| 105.5 | 0.75 | (0.64,0.86) |
| 106.0 | 0.80 | (0.67,0.95) |
| 106.5 | 0.87 | (0.70,1.05) |
| 107.0 | 0.76 | (0.57,0.98) |
| 107.5 | 0.47 | (0.30,0.67) |
| 108.0 | 1.35 | (0.99,1.80) |
| 108.5 | 0.86 | (0.48,1.34) |
| 109.0 | 0.96 | (0.47,1.62) |
| 109.5 | 1.53 | (0.71,2.87) |
| 110.0 | 1.31 | (0.31,3.58) |


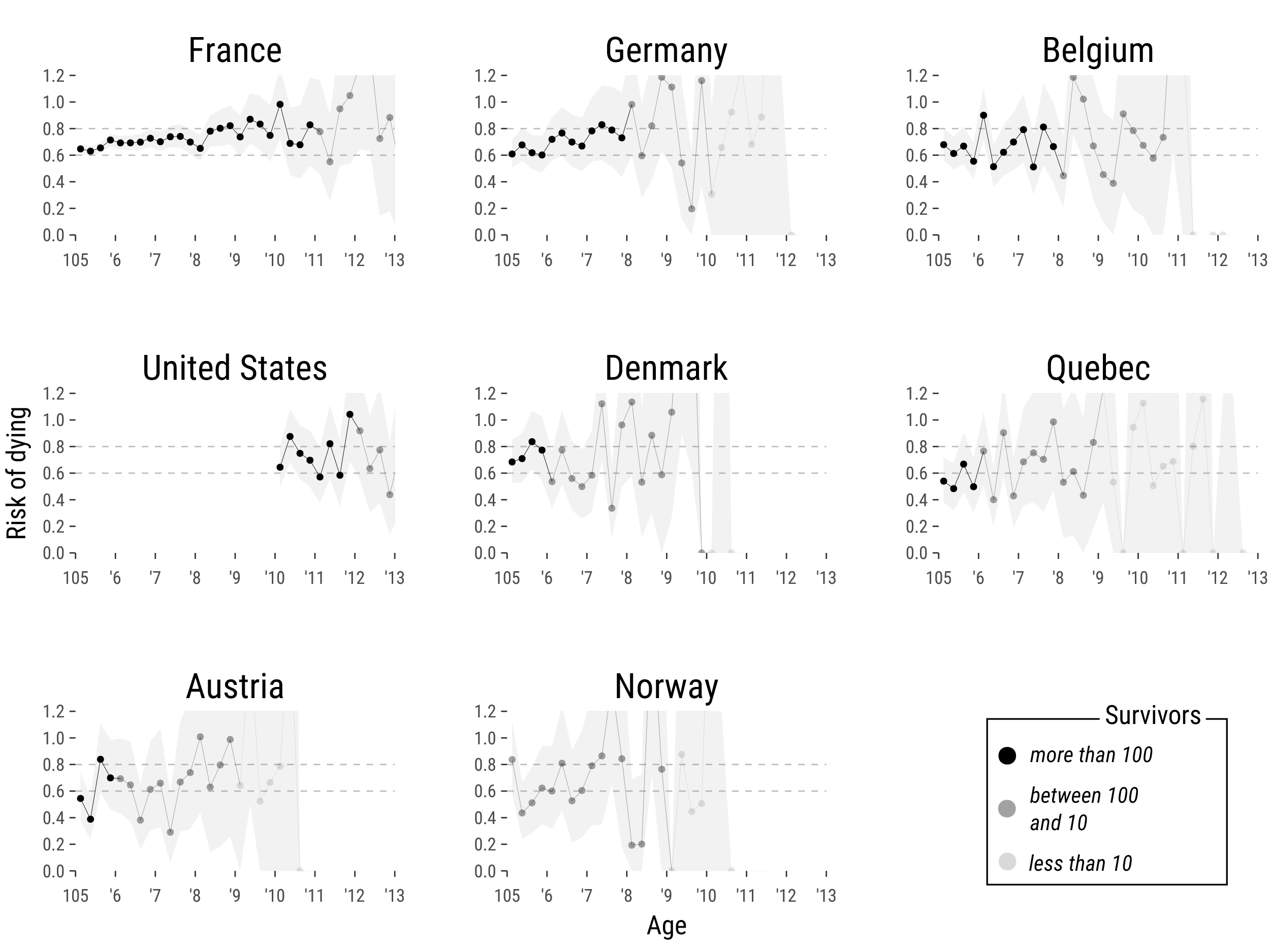


**S1 Fig.** **Risk of dying between ages 105 and 113 with 95% confidence intervals for females**. The risk of dying is expressed in age intervals of three months. The darker the shade of the dot plot, the greater the number of survivors during that specific age (i.e. subjects exposed to the risk of dying).


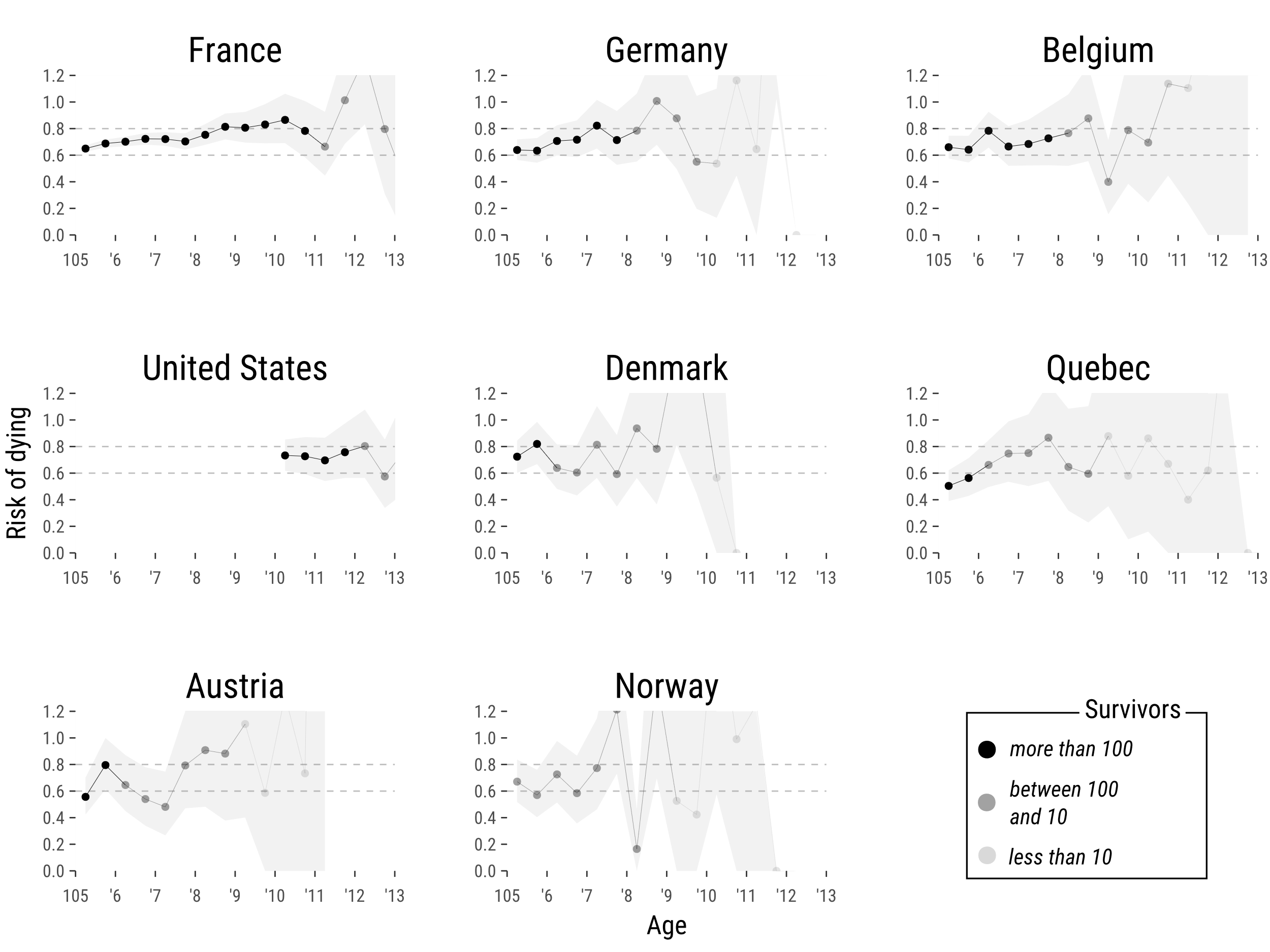


**S2 Fig.** **Risk of dying between ages 105 and 113 with 95% confidence intervals for the total population**. The risk of dying is expressed in age intervals of six months. The darker the shade of the dot plot, the greater the number of survivors during that specific age (i.e. subjects exposed to the risk of dying).

# Coherent patterns between deceleration and levelling off in the risk of dying

In this section we perform additional analyses to examine the concordance of the trajectory of the risk of dying before age 105 with our estimates. Specifically, we calculated the risk of dying between ages 70 and 104 using cohort mortality data from the Human Mortality Database (2021). We retrieved age-specific death counts D(x) and exposures E(x) comprehending the same cohorts and populations included in our analysis in order to enable comparisons.

In Figure S3 we show the calculation of the risk of dying for females in France, Germany and Belgium from ages 70 to 113 using both datasets. Blue circles show estimations from the Human Mortality Database and black points are our non-parametric estimations of the risk of dying from the International Database of Longevity (i.e. same results as in Figure 2 in the manuscript). Note that the estimates in Figure S3 are in log scale.


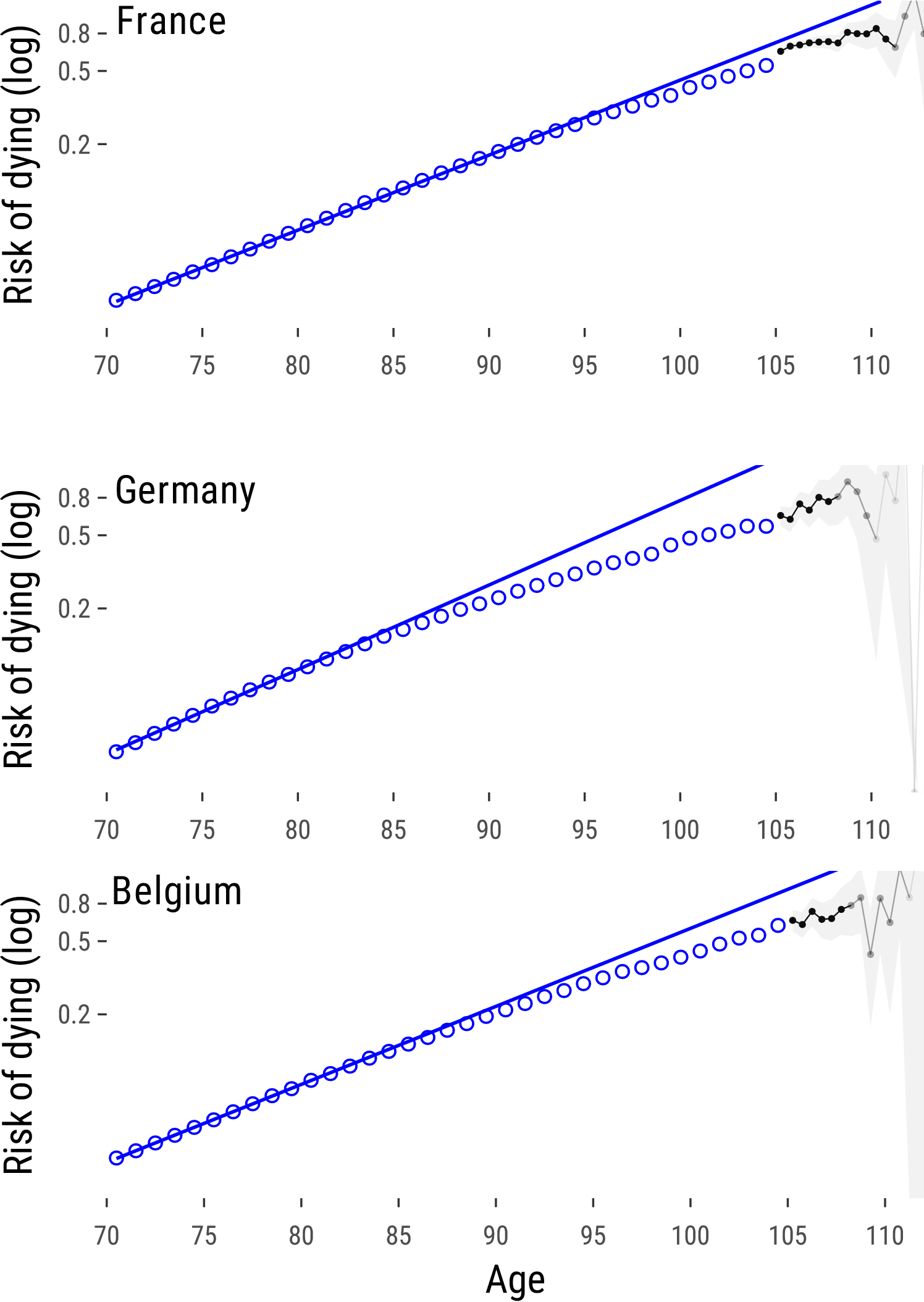


**S3 Fig. Risk of dying from ages 70 to 113 for females.** Estimates from age 70 to age 105 were retrieved from the Human Mortality Database (2021)

Figure S3 shows concordant patterns between both datasets. Moreover, a deceleration in the increase of the risk of dying takes place before age 100. To formally test this pattern, we fitted a Gompertz model from ages 70 and 80 such that the hazard is given by $\mu\left( x \right)=ae^{\mathrm{bx}}$, where the parameter “a” indicates the initial value of the risk of dying and parameter “b” represents the rate of increase in $\mu\left( x \right)$. The estimation was performed by maximizing the Poisson log-likelihood:

$$L\left( x \right)=\sum\left( D\left( x \right)*ln\left( \mu\left( x \right) \right)-E\left( x \right)*\mu\left( x \right) \right)$$

Blue straight lines in Figures S3 show the resulting Gompertz hazard. The parameters of the model are shown in Table S2. From Figure S3, we can observe that the risk of dying deviates from the Gompertz pattern before age 100. Depending on the population such deviations occur at ages between 80 and 90. The findings of this analysis are in line with previous investigations arguing for a deceleration in the risk of dying and a deviation from the Gompertz parametric model occurring prior age 100 (Perks (1932), Horiuchi and Wilmoth (1998)).

**S2 Table. Parameters and goodness of fit for Gompertz hazard model using cohort data from the Human Mortality Database, Females.**

# References

Human Mortality Database. University of California, Berkeley (USA), and Max Planck Institute for Demographic Research (Germany). Available at www.mortality.org or www.humanmortality.de (2021).

D. R. Steinsaltz, K. W. Wachter, Understanding mortality rate deceleration and heterogeneity. Mathematical Population Studies. 13, 19–37 (2006).

M. Finkelstein, V. Esaulova, Asymptotic behavior of a general class of mixture failure rates. Advances in Applied Probability. 38, 244–262 (2006).

C. C. Horvitz, S. Tuljapurkar, Stage dynamics, period survival, and mortality plateaus. The American Naturalist. 172, 203–215 (2008).

Perks, W. (1932). On some experiments in the graduation of mortality statistics. Journal of the Institute of Actuaries (1886-1994), 63(1), 12-57.

Horiuchi, S., & Wilmoth, J. R. (1998). Deceleration in the age pattern of mortality at older ages. Demography, 35(4), 391-412.
